# Development of a double-antigen sandwich ELISA for rapid and accurate detection of antibodies against Capripoxvirus

**DOI:** 10.1128/spectrum.02729-24

**Published:** 2025-05-05

**Authors:** Wanying Wang, Zhengwang Shi, Juncong Luo, Huancheng Liao, Lu Feng, Yuqian Zhu, Yongyu Lin, Xintai Shi, Fan Zhang, Tao Xi, Jie Chen, Hong Tian, Haixue Zheng

**Affiliations:** 1 State Key Laboratory for Animal Disease Control and Prevention, College of Veterinary Medicine, Lanzhou University, Lanzhou Veterinary Research Institute, Chinese Academy of Agricultural Sciences 12426, Lanzhou, Gansu, China; MultiCare Health System, Tacoma, Washington, USA

**Keywords:** Capripoxvirus, 122 protein, DAgS-ELISA, antibody detection

## Abstract

Lumpy skin disease, goatpox, and sheeppox have rapidly spread in many countries, leading to the emergence of new mutant strains that have caused substantial damage and posed significant threats to animal husbandry in endemic areas. Effective surveillance and disease control necessitate a simple and precise diagnostic method for detecting antibodies against Capripoxvirus (CaPV). In the present study, a double-antigen sandwich enzyme-linked immunosorbent assay (DAgS-ELISA) based on the 122 protein expressed early in viral replication and with high immunogenicity and conservation was developed to detect antibodies against lumpy skin disease virus (LSDV), goatpox virus (GTPV), and sheeppox virus (SPPV). The receiver operating characteristic (ROC) was performed to identify the best threshold value using 109 negative and 57 positive samples. The established test exhibited an area under the curve (AUC) of 0.997, and the test achieved a diagnostic sensitivity of 94.7% and a specificity of 96.3% when the S/P value threshold was set at 0.304. This method can detect the positive standard serum at a dilution of 1:32. The performances of the DAgS-ELISA were highly consistent with the commercial kit in testing 150 clinical serum samples, which were very similar (kappa value = 0.759). Moreover, the analysis of antibody dynamics revealed that the results exhibited higher sensitivity in early detection of total antibodies compared with the commercial kit. In conclusion, the DAgS-ELISA method demonstrated excellent repeatability, user-friendliness, and the ability to provide early detection of CaPV antibodies, which is highly suitable for epidemiological studies and evaluation of vaccine-induced antibody responses.

**IMPORTANCE:**

In this study, a double-antigen sandwich ELISA was developed based on the 122 protein with high immunogenicity and conservation during early viral replication, aiming to detect antibodies against Capripoxvirus. This method features high sensitivity and specificity, is cost-effective, simple, reproducible, and suitable for extensive testing. It can detect antibodies at an early phase and serves as a powerful tool for epidemic monitoring, prevention, and control.

## INTRODUCTION

The genus *Capripoxvirus* (CaPV) includes lumpy skin disease virus (LSDV), goatpox virus (GTPV), and sheeppox virus (SPPV), all of which belong to the vertebrate poxvirus subfamily of Poxviridae (1–3). These viruses can cause lumpy skin disease, goatpox, and sheeppox, respectively, in domestic animals (4). CaPV infections are highly contagious and result in the development of variable-sized nodules and edema on the skin and organ surfaces of goats, sheep, and cattle (5–7). This pathological state not only significantly reduces the quality of cattle and sheep hides but also has a serious impact on the production performance and economic value of cattle and goats, which in turn poses a great threat to and destroys the entire cattle, goat, and sheep farming industry (8–10).

CaPV is characterized by its linear double-stranded DNA with a remarkably similar genomic structure, displaying a 96%–97% nucleotide similarity among its members (11–14). Moreover, CaPV does not exhibit strict host specificity (15–17). Therefore, it is difficult to distinguish the three viruses of the genus Capripoxvirus on the basis of host and clinical signs. Current diagnostic methods mainly include the virus neutralization test (VNT), indirect fluorescent antibody test (IFA), and enzyme-linked immunosorbent assay (ELISA) (18–24). However, VNT requires the manipulation of live viruses and is time-consuming, and IFA is time-consuming and has poor specificity, and both have biosafety issues. Among these methods, ELISA stands out as the most convenient and cost-effective serological diagnostic technique, making it suitable for high-throughput screening. There are few ELISAs for antibodies against CaPV, so it is necessary to develop a highly specific, sensitive, convenient, and accurate diagnostic method to detect antibodies against CaPV.

The ORF122 gene is located within the central coding region (ORF24–ORF123) of GTPV, which includes genes involved in viral DNA replication, construction, and assembly of viral particles, and is highly conserved and genetically stable (25). As a component of the CaPV capsule membrane, the 122 protein demonstrates significant antigenicity and immunogenicity (10). Previous studies have shown that rabbits immunized with recombinant SPPV-122 protein can produce antibodies with virus-neutralizing activity (26). Furthermore, the full-length ORF122 gene is relatively short and lacks specific structures (27). Similar to P32, the main immunodominant protein of CaPV (28), the 122 protein is also suitable for diagnosing CaPV infections and can be more easily expressed in prokaryotic systems while maintaining its robust biological activity. Therefore, the 122 protein holds great clinical value and shows promising applications in diagnosing CaPV infections as well as vaccine research. In this study, we utilized the immunodominant CaPV protein 122 as the solid-phase antigens in ELISA while using horseradish peroxidase (HRP)-labeled 122 as an enzyme-labeled antigen. By this method, a DAgS-ELISA with high sensitivity and specificity was established. The DAgS-ELISA substantially reduced the occurrence of false-positive results (29), enabling the identification of total antibodies, including IgG, IgM, and other immunoglobulins (30, 31), and it is particularly advantageous for early diagnosis (32), providing technical support for monitoring CaPV antibodies and evaluating immune responses.

## MATERIALS AND METHODS

### Experimental materials

Positive sera of LSDV, GTPV, SPPV, foot-and-mouth disease virus type O (FMDV-O), Orf virus (ORFV), *Brucella*, bovine viral diarrhea virus (BVDV), as well as negative sera of LSDV, GTPV, and SPPV, along with 109 CaPV negative sera, 57 CaPV positive sera, 150 CaPV (goats, 20; sheep, 35; and cattle, 95) clinical samples and the antibody dynamics sera from the LSDV-inactivated vaccine were all archived at the Lanzhou Veterinary Research Institute, Chinese Academy of Agricultural Sciences, China (Table 1). *Escherichia coli* BL21 (DE3) receptor cells, β-D-1-thiogalactopyranoside and the Wellwash plate washer used were purchased from ThermoFisher Scientific Inc. The HRP labeling kit and the small ubiquitin-related protein (SUMO) were purchased from Sigma Aldrich (Shanghai) Trading Co. The ID Screen Capripox Double Antigen Multi-species was produced from IDVet, Grabels, France. The fully automated enzyme labeler used for plate reading was purchased from Bio-Tek, Inc.

**TABLE 1:** Description and quantity of serum samples

Sera	Positive/negative	Number
FMDV-O	Negative sera	1
ORFV		1
*Brucella*		1
BVDV		1
LSDV		1
GTPV		1
SPPV		1
LSDV	Positive sera	1
GTPV		1
SPPV		1
CaPV	Negative sera	109
	Positive sera	57
CaPV clinical sera	Unknown	95 (LSDV)
		20 (GTPV)
The antibody dynamics in sera	Unknown	35 (SPPV)
		36

### Homology analysis of 122 gene sequence

A total of 17 strains of CaPV, 4 strains of GTPV, 9 strains of LSDV, and 4 strains of SPPV were selected for homology analysis of 122 gene sequences. The 122 protein sequence (GenBank ID: OP508345.1) transmembrane helices were predicted by TMHMM-2.0, and the extracellular region was captured for expression.

### Construction and expression of recombinant 122 (r122) protein

Based on the structure prediction of the 122 protein, the non-transmembrane region of the 122 protein (position 68–196 of the amino acid region) was selected for protein expression. This segment was cloned into the pET-SUMO vector, which was produced by GeneCreate Biological Engineering Co., Ltd. (Wuhan, China). The recombinant plasmid pET-SUMO-122 was transformed into *E. coli* BL21 (DE3) receptor cells. A single clone was selected and cultivated, and protein expression was induced using isopropyl β-D-1-thiogalactopyranoside. Bacteria were harvested through centrifugation, resuspended in PBS, and subsequently lysed by ultrasonication. After centrifugation, the supernatant and precipitate were collected, and the forms of expressed target protein were analyzed using sodium dodecyl sulfate polyacrylamide gel electrophoresis (SDS-PAGE).

### Purification and enzyme digestion of r122 protein

Once the optimal conditions for protein expression were determined, large-scale production of the target protein was initiated and followed by purification via nickel column chromatography. The purified r122 protein was analyzed using SDS-PAGE to verify the purification of the target protein, and its reactivity with CaPV positive and negative sera was identified by Western blotting. Subsequently, the SUMO protease and r122 that have been dialyzed by 25 mM Tris-HCl were mixed with at a ratio of 10U:1 mg. After incubation at 4°C for 16 h, the mixture buffer was purified by Ni^2+^ affinity chromatography. The tag SUMO was removed by Ni^(2+)^-chelating chromatography, and the 122 protein was obtained by the effluent of the digestion mixture.

### HRP tagging of the r122 protein

The r122 protein was labeled with HRP according to the instructions of the HRP labeling kit (labeled protein was named HRP-122). After labeling, the solution was loaded onto a dialysis card, dialyzed with 0.1 M and pH 7.4 phosphate-buffered saline (PBS), and then subjected to centrifugation at 10,000 rpm for 30 min. Subsequently, the supernatant was collected, aliquoted, and stored at −80°C.

### Establishment and optimization of the DAgS-ELISA method

In order to establish and optimize the DAgS-ELISA method, coating concentration of the r122 protein and dilution of HRP-122 were determined by checkerboard titration. The 122 protein was coated at 1, 2, and 3 µg/mL in ELISA plates overnight at 4°C, and the plates were blocked overnight at 4°C with 5% BSA. After washing four times with PBST, the CaPV-positive sera and the CaPV-negative sera were used to determine the optimal conditions. Then, 50 µL of sera sample was added to each well, incubated at 37°C for 30 min, and the plate was washed. HRP-122 was diluted at a dilution of 1:40,000, 1:60,000, and 1:80,000, respectively, and 50 µL was added to each well, incubated at 37°C for 30 min, and the plate was washed. Subsequently, 50 µL of 3,3′,5,5′-Tetramethylbenzidine (TMB) was added to each well and incubated at 4°C for 12 min away from light. Finally, the reaction was terminated with 50 µL of 2M H_2_SO_4_ per well. The reaction conditions at the maximum P/N (positive sample mean OD/negative control mean OD) value were determined as the optimal antigen coating concentration and the optimal dilution of HRP-122, and then the optimal sera dilution was determined.

### Determination of cut-off value

Under the optimized ELISA conditions, 109 negative sera samples and 57 positive sera samples were detected, and the S/P value of each result was calculated, S/P = (sample mean OD – negative control mean OD)/(positive control mean OD − nnegative control mean OD) (33). Then, receiver operating characteristic (ROC) curves were plotted and analyzed for diagnostic sensitivity and specificity based on S/P values. The Youden index was calculated according to the formula Youden index = sensitivity + specificity – 1, and the S/P value corresponding to the maximum Youden index is the cutoff value (34, 35).

### Diagnostic specificity, sensitivity, repeatability evaluation

The DAgS-ELISA was conducted to detect the presence of antibodies against LSDV, GTPV, SPPV, *Brucella*, ORFV, FMDV, and BVDV in the sera. For each sample of LSDV, GTPV, and SPPV negative sera, three wells were utilized for parallel testing. LSDV, GTPV, and SPPV positive sera were diluted at a ratio of 1:2 to 1:128 and subjected to DAgS-ELISA under optimized conditions to assess the sensitivity of the method. To assess the reproducibility of the DAgS-ELISA, sera of known background (four each of positive and negative sera) were selected. The reproducibility of the method was analyzed by calculating the coefficient of variation for intra-batch replicates with five parallel wells in one ELISA plate and inter-batch replicates with two parallel wells in each of the three different batches of ELISA plates.

### Evaluation of antibody dynamics

The animal numbers used in this study were selected based on established practices in the field, as demonstrated by Zhugunissov (36), who immunized 15 cattle divided into three groups for safety evaluation of the goatpox virus (G20-LKV) vaccine strain. In our study, cattle testing negative for Capripoxvirus antibodies using the ID Screen Capripox Double Antigen ELISA kit were selected as experimental animals. Five cattle were immunized with inactivated LSDV vaccine, alongside one control animal. Serum samples were collected from all animals at 0, 7, 14, 21, 28, and 35 days post-immunization to monitor antibody responses. Antibody levels in all sera samples were detected and analyzed, and antibody extinction curves were plotted. Concurrently, the same samples were tested using the commercial kit for comparison.

### Consistency analysis of DAgS-ELISA with commercialized kits

A total of 150 clinical sera samples were simultaneously tested using the ID Screen Capripox Double Antigen Multi-species and DAgS-ELISA kits. The results were analyzed to compare the agreement between the two methods.

## RESULTS

### Homology analysis of 122 genes

**Fig 1:**
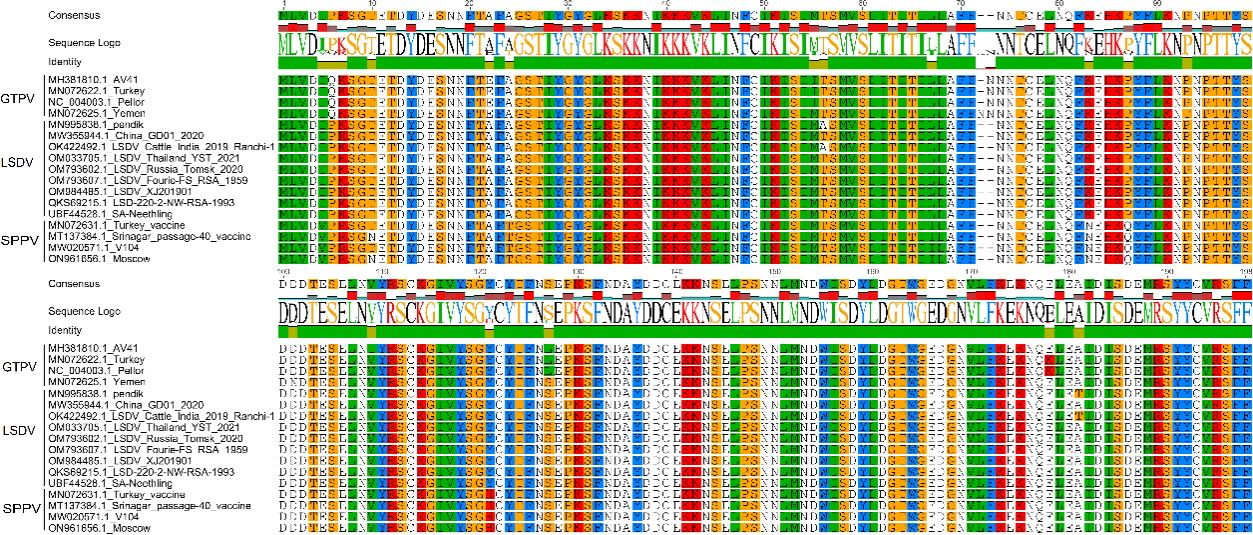
Homology analysis of 122 genes from 17 CaPV strains, including 4 strains of GTPV, 9 strains of LSDV, and 4 strains of SPPV. Comparative analysis of the gene sequences showed that the homology between the 17 strains of GTPV, LSDV, and SPPV was not less than 93.4%.

**Fig 2:**
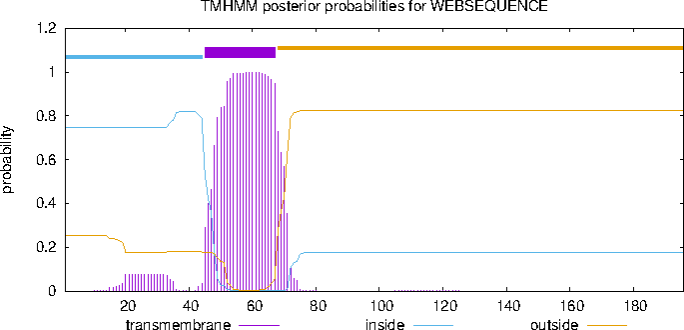
The 122 protein structure prediction. Based on the structure prediction of the 122 protein, the non-transmembrane region of the 122 protein (position 68–196 of the amino acid region) was selected for protein expression.

Comparative analysis of the gene sequences showed that the homology between the 17 strains of GTPV, LSDV, and SPPV was not less than 93.4% (Fig. 1), confirming the high conservation of the 122 gene. Based on the structure prediction of the 122 gene (Fig. 2), the extracellular region was selected for expression.

### Protein expression, purification, and characterization

**Fig 3:**
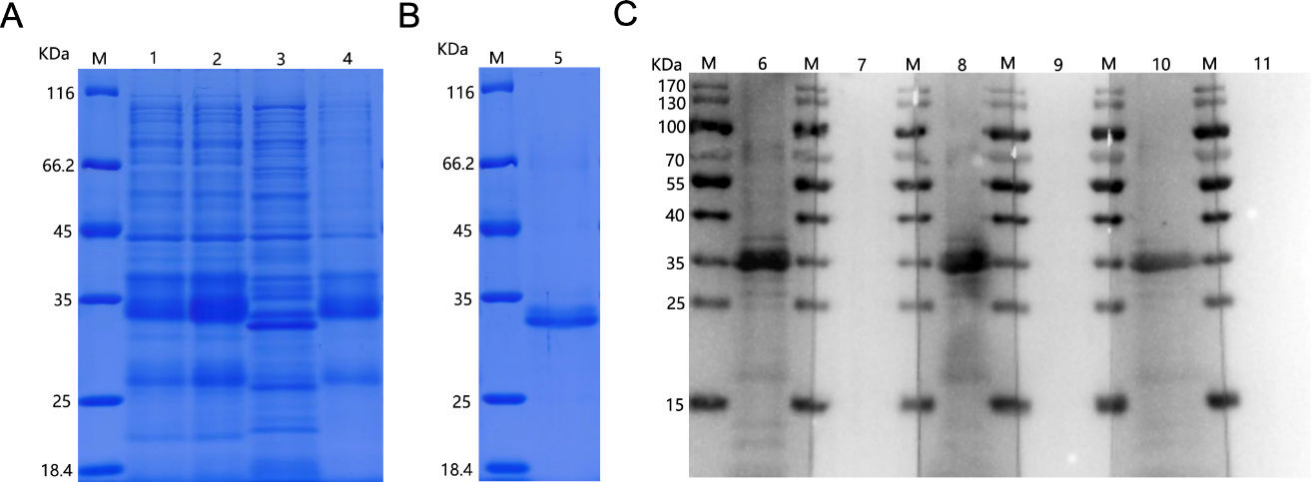
Expression, purification, and identification of the 122 protein. (**A**) 122 protein expression. M, protein marker; 1, uninduced *E. coli* BL21 (DE3) culture; 2, induced *E. coli* BL21 (DE3) culture; 3, induction of the soluble fraction of *E. coli* BL21 lysate; 4, induction of precipitated fraction of *E. coli* BL21 lysate. (**B**) 122 protein purification. M, protein marker; 5, purified 122 protein. (**C**) Western blot identification of purified 122 protein. CaPV positive and negative sera at a dilution of 1:200 were used as primary antibodies. M, protein marker; 6, LSDV positive serum; 7, LSDV negative serum; 8, GTPV positive serum; 9, GTPV negative serum; 10, SPPV positive serum; 11, SPPV negative serum.

SDS-PAGE analysis indicated that the r122 protein had an approximate molecular weight of 26 kDa (Fig. 3A and B). Western blotting demonstrated strong reactivity between the purified r122 protein and CaPV positive sera (Fig. 3C).

### Establishing and optimizing the DAgS-ELISA

**Fig 4:**
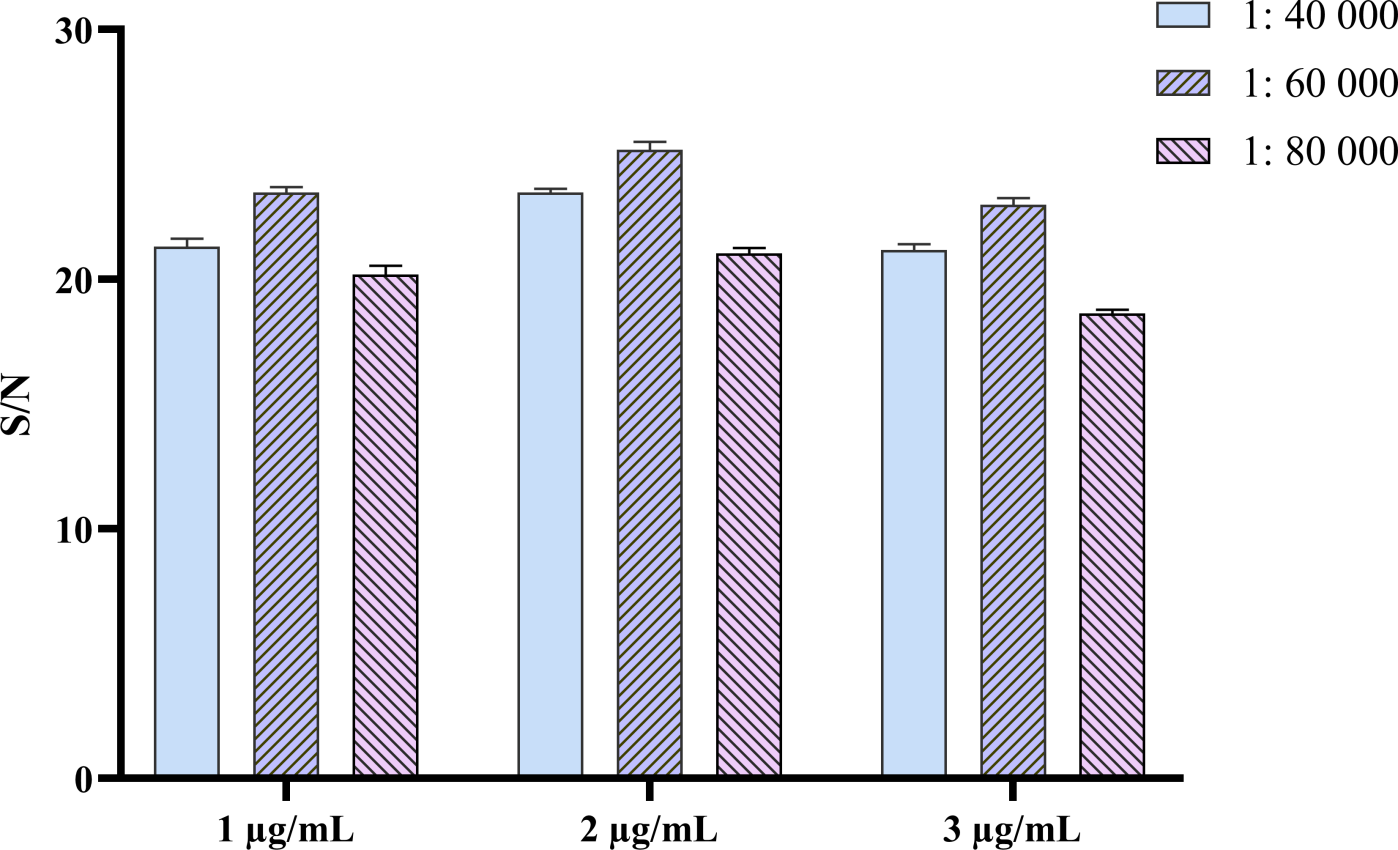
Determination of optimal antigen-coating concentration and optimal dilution of enzyme-labeled antigen.

Statistical analysis of the results showed that the P/N values were greatest when the concentration of encapsulated antigen and the dilution ratio of enzyme-labeled secondary antigen were 2 µg/mL and 1:60,000, respectively (Fig. 4). Using this reaction condition as the optimal reaction condition, the optimal dilution ratio of sera was determined to be 1:2, and the optimal incubation time of serum and enzyme-labeled antigen was determined to be 30 min.

### Determination of cutoff values

**Fig 5:**
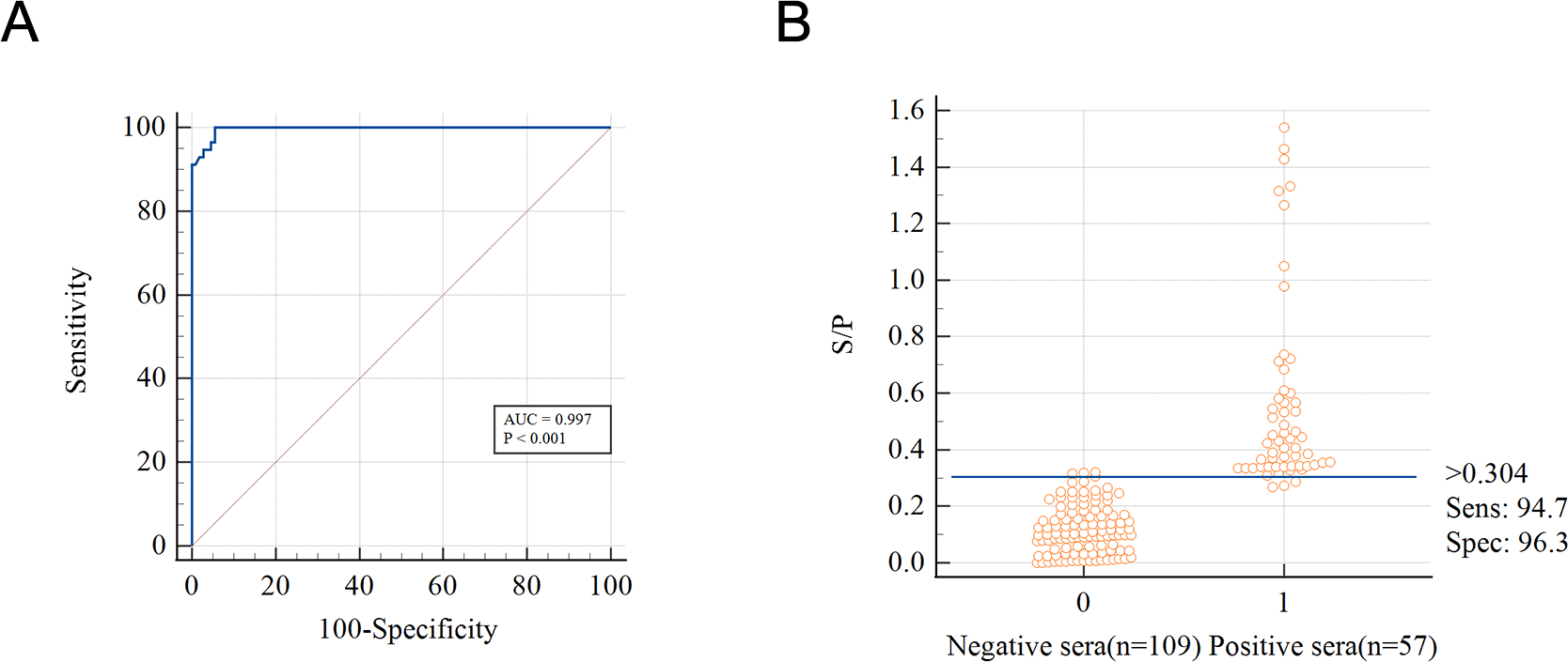
Determination of cutoff values for a double-antigen sandwich ELISA based on CaPV 122 protein. (**A**) ROC analysis of CaPV-negative samples (*n* = 109) and CaPV-positive samples (*n* = 57). (**B**) A dynamic dot plot diagram illustrating the serum sample’s S/P value with a cut-off value of 0.304.

The DAgS-ELISA method was evaluated by testing 166 samples of serum, consisting of 109 negative samples and 57 positive samples. Then, the ROC curve of the S/P data were analyzed, and the optimal cut-off value was calculated according to the analysis results. The established test had an area under the curve (AUC) of 0.997 according to the ROC analysis (Fig. 5A). With a cut-off value of 0.304, the Jorden index is the highest, the diagnostic sensitivity is 94.7%, and the specificity is 96.3% (Fig. 5B).

### Diagnostic specificity, sensitivity, and repeatability evaluation

**Fig 6:**
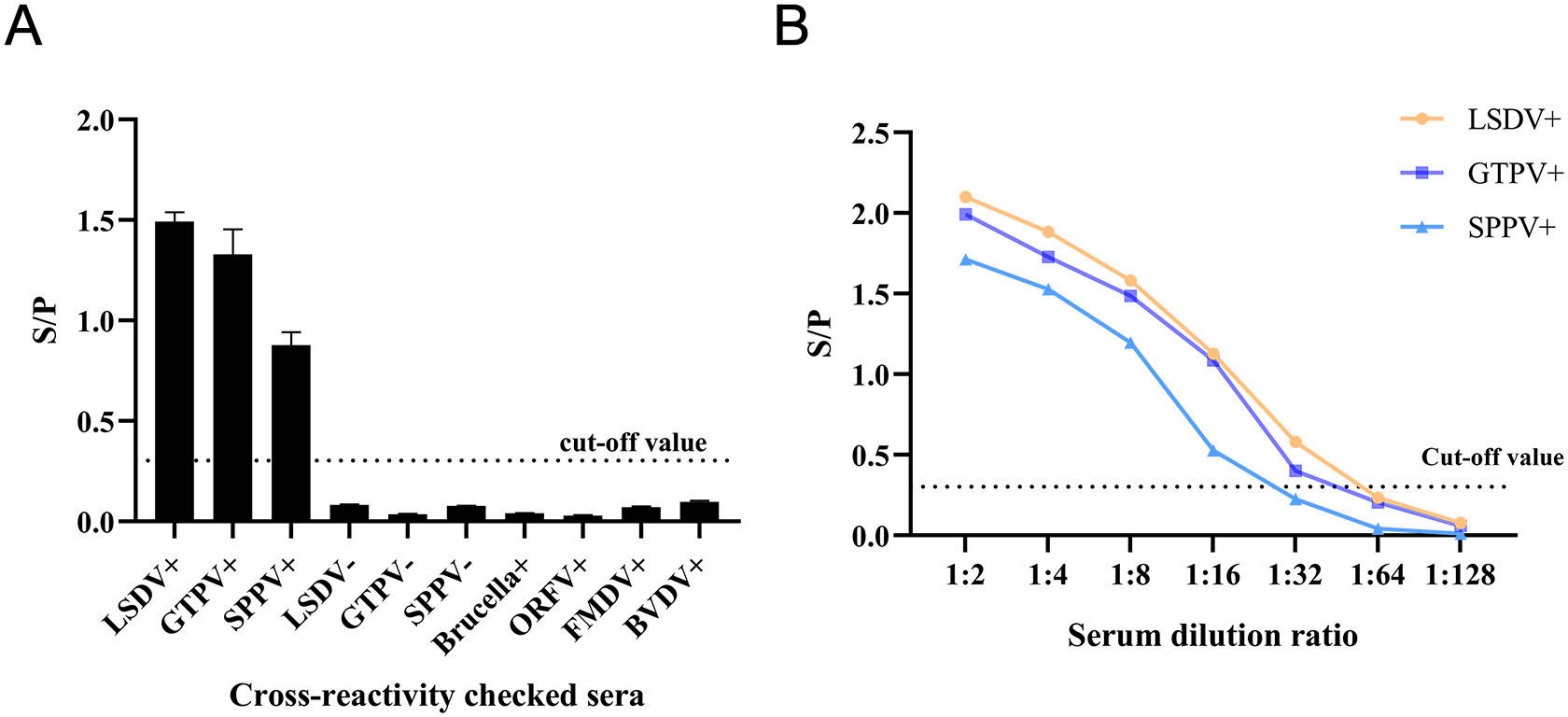
Method evaluation for a doubleantigen sandwich ELISA based on Capv 122 protein. (**A**) Specificity analysis of DAgS-ELISA. (**B**) Sensitivity analysis of the DAgS-ELISA.

The results of the specificity test showed good specificity without cross-reactivity with positive sera of *Brucella*, ORFV, FMDV, and BVDV (Fig. 6A). Sensitivity tests found the method to be sensitive up to 1:32 for LSDV and GTPV and 1:16 for SPPV (Fig. 6B), indicating that this DAgS-ELISA has good sensitivity. Repeatability tests revealed that the intra-assay and inter-assay coefficients of variation of the samples were less than 10%(Table 2), demonstrating that the DAgS-ELISA method has good reproducibility.

**TABLE 2:** Repeatability assay results for DAgS-ELISA

Sample no.		Intra-assay		Inter-assay	
		X ± SD	Cv %	X ± SD	Cv %
Positive samples	1	1.928 ± 0.045	2.32	1.916 ± 0.047	2.43
	2	1.848 ± 0.054	2.92	1.808 ± 0.038	2.08
	3	1.984 ± 0.047	2.36	1.931 ± 0.079	4.07
	4	1.379 ± 0.019	1.35	1.348 ± 0.051	3.82
Negative samples	5	0.098 ± 0.004	3.64	0.117 ± 0.009	7.77
	6	0.109 ± 0.005	4.41	0.111 ± 0.004	3.28
	7	0.121 ± 0.004	3.35	0.195 ± 0.017	8.82
	8	0.103 ± 0.005	4.77	0.100 ± 0.008	7.62

### Assessment of antibody dynamics

**Fig 7:**
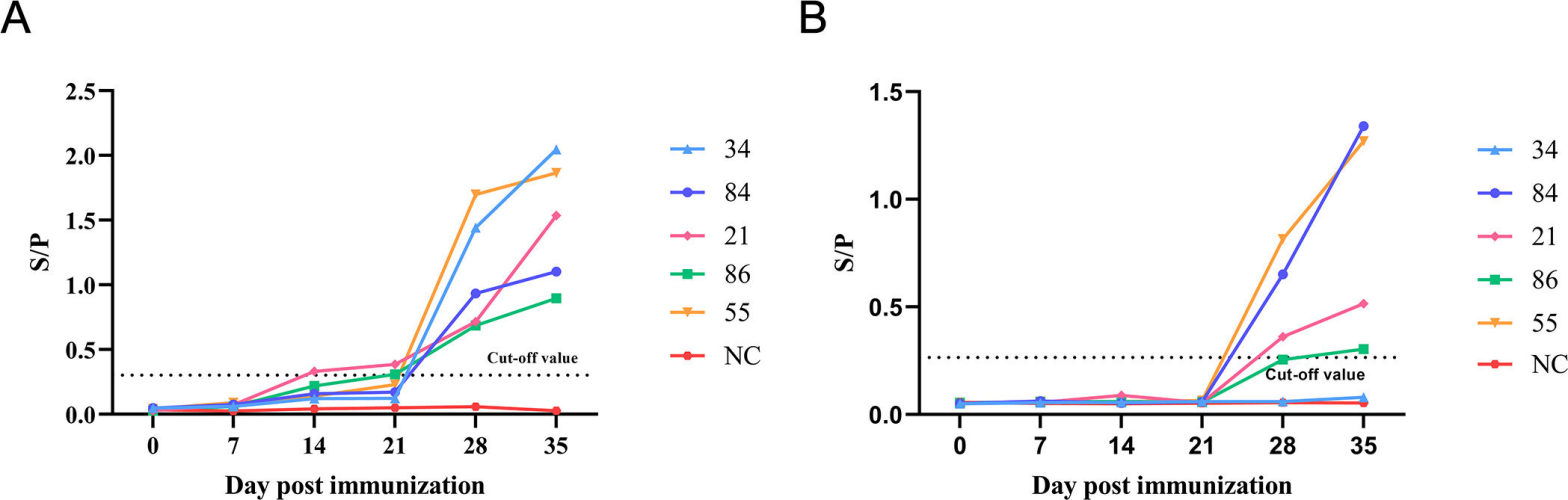
Dynamic evaluation of LSDV antibodies in five immunized cattle. (**A**) The detection results of the DAgS-ELISA. (**B**) The detection results of the commercial kit.

Two methods were used to conduct a follow-up study on the antibody levels of the test cattle. The results of the DAgS-ELISA assay showed antibody responses in some animals from days 14 to 21 and positive responses in all immunized animals by day 28, and maintained these responses until day 35 (Fig. 7A). The IDVet commercial kit demonstrated limited detection sensitivity, failing to identify antibody responses before day 21. While it produced slightly different results from the established method after day 21 (Fig. 7B), its performance was notably inferior in specific cases. A striking example is the monitoring of bovine serum number 34. The established method successfully detected elevated antibody responses between 21 and 28 days, whereas the commercial kit failed to detect any antibodies during this period (Fig. 7A and B).

### Consistency analysis of DAgS-ELISA with commercial kit

The DAgS-ELISA and the IDVet commercial kit were used to test 150 clinical sera samples. After statistical analysis of the data, a Kappa value of 0.759 was calculated, indicating a high degree of consistency between the two assays(Table 3).

**TABLE 3:** Consistency analysis of DAgS-ELISA with commercial kit^*a*^

DAgS-ELISA	The ID Screen Capripox Double-Antigen Multi-species		
	Positive	Negative	Total
Positive	72	10	82 (54.7%)
Negative	8	60	68 (45.3%)
Total	80 (53.3%)	70 (46.7%)	150

^*a*^ Po = (72 + 60)/150 = 0.88; Pe = 0.799*0.794 + 0.201 × 0.206 = 0.503. K= (0.88 − 0.503)/(1 − 0.503) = 0.759. Kappa formula for k = (Po − Pe)/(1 − Pe), kappa calculation results for −1–1, but usually kappa falls between 0 and 1 and can be divided into five groups to indicate the consistency of the different levels: 0.0–0.20, very low consistency, slight; 0.21–0.40, fair; 0.41–0.60, moderate; 0.61–0.80, substantial; and 0.81–1, almost perfect.

## DISCUSSION

LSD, GTP, and SPP have had a profound impact on the trade and industrial development of cattle and sheep and their products (7). CaPV viruses exhibit significant genetic similarity among their various strains and have the potential for cross-infection (12). Therefore, the establishment of an early, rapid, and accurate method for the detection of antibodies against CaPV is crucial for the prevention and control of the disease. Currently, the total number and diversity of CaPV epidemic strains in China remain unknown (27).

Furthermore, the classification of these strains as either natural epidemic strains, vaccine-derived strains, or recombinant strains remains uncertain, presenting a challenge to the management of CaPV in China (27). Several serological diagnostic methods for CaPV diagnosis are currently in use. The DAgS-ELISA method is based on the formation of conjugates of the antibody to be tested with solid-phase antigens and enzyme-labeled antigens, which employs two specific antigens conjugated to an antibody to enable the detection of different types of antibodies in a sample while avoiding the interference of non-specific antibodies, thus reducing the generation of false-positive results. Consequently, the DAgS-ELISA method enhances specificity and sensitivity, resulting in more efficient use of time and resources, increased convenience, and reduced risk of cross-contamination (37, 38).

The selection of specific antigens is crucial for ELISA. Numerous studies on poxviruses have confirmed the essential role of extracellular enveloped virus (EEV) vesicle membrane proteins in poxvirus immunity (26, 27). The 122 protein, which serves as a vesicle membrane component of EEV (19), exhibits genetic conservation with a concise full-length structure and simple architecture that facilitates easy expression (20). Research has demonstrated that deletion of the transmembrane domain does not affect the natural antigenic site of the 122 protein, while still maintaining its robust biological activity, thereby facilitating further research and development (20). Moreover, the 122 protein demonstrates favorable immunogenicity. The DAgS-ELISA test revealed no cross-reactivity with other common pathogens in cattle and sheep, thus confirming the strong specificity of the 122 protein.

Currently, the only diagnostic kit that can detect all three CAPVs (LSDV, GTPV, and SPPV) is the commercial kit produced by IDVet. According to the comparison of clinical serum diagnosis, the DAgS-ELISA established in this study has a high consistency with IDVet’s commercial kits (kappa value = 0.759), but there are still differences in the detection of a small number of serum samples. In order to analyze the differences between the DAgS-ELISA established in this study and commercial kits, we performed antibody dynamics analysis of immunized bovine. In monitoring the antibody dynamics of immunized cattle, the DAgS-ELISA assay demonstrated superior early detection capability. As early as day 7 to day 21, the immunized animals showed a low antibody response, but only a small number of animals showed a positive antibody response at day 21. Positive responses were consistently maintained in all immunized animals from day 28 to day 35. In contrast, the IDVet commercial kit failed to detect antibodies before day 21. The early detection by DAgS-ELISA at day 7 primarily reflects IgM production during the initial immune response, indicating the assay’s ability to detect total antibodies at earlier stages. From day 7 onwards, DAgS-ELISA results demonstrated a gradual increase in total antibody levels, which remained stable between days 14 and 21. After day 21, a significant shift in antibody composition was observed, characterized by decreasing IgM levels and a concurrent increase in IgG production, resulting in elevated total antibody levels. Interestingly, antibodies from one of the cattle were never detected by commercial kits, but the ELISA method established in this study was effective. We suspect that this may be due to differences in individual immune responses to which commercial kits are not sensitive, which partly explains the different results of the two methods in some clinical samples. By analyzing the trend in antibody dynamics, it becomes evident that DAgS-ELISA primarily detects various types of antibodies including IgM and IgG with high sensitivity and specificity for early detection purposes. These findings underscore the advantages offered by DAgS-ELISA in antibody detection and its suitability for evaluating clinical immunization effects. However, this method currently only detects total antibodies, and further research is needed to distinguish between recent and distant infections.

The high specificity of our assay (96.3%) is particularly significant given the substantial prevalence of Capripoxvirus (CaPV) infections in endemic regions. Epidemiological studies have demonstrated considerable infection rates, with Limon (39) reporting incidence risks of 33% in cattle, 53% in sheep, and 50% in goats in Nigeria. Furthermore, Suresh (40) documented seroprevalence rates of 44% in Asia and 16% in Africa. These prevalence levels, combined with the limitations of current diagnostic methods (including immunofluorescence, immunoprecipitation, VNT, PCR, and ELISA) in accurately detecting and differentiating CaPV strains, underscore the importance of our newly developed method. The assay’s specificity is therefore well-suited for epidemiological studies and evaluation of vaccine-induced antibody responses, as the relatively high prevalence rates in endemic regions mitigate potential concerns regarding false positive results. The present study successfully established a DAgS-ELISA, which demonstrated no cross-reactivity with *Brucella*, ORFV, FMDV, and BVDV. It exhibited a sensitivity of 1:32, and the intra-assay and inter-assay coefficients of variation were less than 10%. Compared with commercially available kits, this novel DAgS-ELISA method performed well in the detection of antibodies against CaPV (kappa value = 0.759), making it suitable for high-throughput testing of clinical sera samples. This method provides both a theoretical basis and data support for the development of scientifically sound immunization protocols for large-scale cattle and sheep farms. The assay is cost-effective, simple, reproducible, and suitable for large-scale testing, thus providing an effective means for preventing and controlling CaPV infection transmission while serving as a valuable tool for monitoring CaPV antibodies and conducting epidemiological investigations.

## Data Availability

The data that support the findings of this study are available from the corresponding author upon reasonable request.
